# An integrated classification of tumor suppressor *IKZF1* inactivation and oncogenic activation in Philadelphia chromosome‐like acute lymphoblastic leukemia

**DOI:** 10.1002/hem3.82

**Published:** 2024-05-21

**Authors:** Zicong Huang, Ling Zhang, Xiaoyuan Gong, Jia Li, Shiyu Deng, Zihong Cai, Bingqing Tang, Kangyu Huang, Xin Li, Weihua Zhao, Yang Xu, Li Xuan, Qifa Liu, Ying Wang, Suning Chen, Hongsheng Zhou

**Affiliations:** ^1^ Department of Hematology, Nanfang Hospital Southern Medical University Guangzhou China; ^2^ Guangdong Provincial Clinical Research Center for Hematologic Diseases, Nanfang Hospital Southern Medical University Guangzhou China; ^3^ Department of Hematology Ganzhou People's Hospital (Nanfang Hospital Ganzhou Hospital) Ganzhou China; ^4^ National Clinical Research Center for Hematologic Diseases, Jiangsu Institute of Hematology The First Affiliated Hospital of Soochow University Suzhou China; ^5^ State Key Laboratory of Experimental Hematology Institute of Hematology and Blood Diseases Hospital, Chinese Academy of Medical Sciences & Peking Union Medical College Tianjin China; ^6^ Department of Hematology, The Third Xiangya Hospital Central South University Changsha China; ^7^ Department of Hematology The First Affiliated Hospital of Guangxi Medical University Nanning China

## Abstract

Philadelphia chromosome (Ph)‐like acute lymphoblastic leukemia (ALL) is recognized for its genetic and clinical diversity. In this study, we identified a novel high‐risk subset of Ph‐like ALL, characterized by the activation of oncogenic signaling and the inactivation of the tumor suppressor gene *IKZF1*, resulting in a dismal outcome. The association between cytogenetic aberrations and clinical features was assessed on a cohort of 191 patients with Ph‐like ALL. Our findings revealed that patients with inactivation of *IKZF1* combined with activation of oncogenic signaling (*CRLF2/EPOR/JAK2* rearrangements or *p‐CRKL/p‐STAT5* high expression) had the worst outcome (3‐year overall survival [OS] of 28.8% vs. 80.1% for others, *p* < 0.001; 2‐year event‐free survival [EFS] of 6.5% vs. 57.0% for others, *p* < 0.001). Multivariable analysis demonstrated that this high‐risk feature was an independent inferior prognostic factor (adjusted hazard ratio for OS = 4.55, 95% confidence interval [CI]: 2.35–8.81, *p* < 0.001; adjusted hazard ratio for EFS = 3.27, 95% CI: 1.99–5.39, *p* < 0.001). Allogeneic hematopoietic stem cell transplantation was associated with improved prognoses in patients within the high‐risk subgroup. In conclusion, this study identified a clinically distinct entity that possesses effective prognostic features and provides potential guidance for refining risk stratification in Ph‐like ALL.

## Graphical abstract

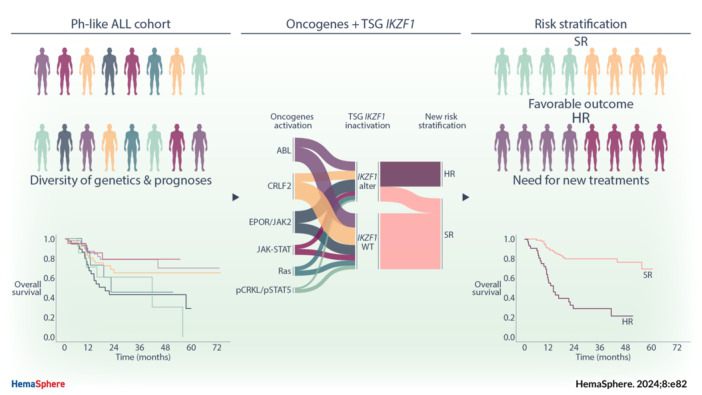

## INTRODUCTION

Philadelphia chromosome (Ph)‐like acute lymphoblastic leukemia (ALL) is a genetically and clinically heterogeneous disease characterized by various chromosomal rearrangements, sequence mutations involving kinase or cytokine receptor signaling pathway activation and a high frequency of alterations in transcription factors involved in B cell development, including *IKZF1*, *PAX5*, and *EBF1*.^1^, ^2^, ^3^ Despite improvements in outcomes with tyrosine‐kinase inhibitors (TKIs) for patients with ABL‐class Ph‐like ALL,^4^ the overall prognosis for Ph‐like ALL remains inferior, with a 5‐year event‐free survival (EFS) rate of 22.5% and an overall survival (OS) rate of 23.8%, compared to 50%–60% OS observed in other subsets of Ph‐negative (Ph^−^) ALL.^3^ Therefore, it is crucial to identify the subsets with the worst outcomes within the highly diverse landscape of Ph‐like ALL to develop risk‐directed strategies. The oncogenic pathways involved in Ph‐like ALL predominantly comprise the ABL‐class, *CRLF2*, and JAK‐STAT signaling, which are associated with pro‐proliferative or anti‐apoptotic properties and are part of the current risk classification.^5^, ^6^ Moreover, tumor suppressor gene (TSG) *IKZF1* deletions have emerged as the most important cytogenetic aberrations in B‐cell ALL (B‐ALL). They are frequently observed in approximately 50%–60% of Ph‐like ALL cases. Emerging evidence has shown that *IKZF1* aberrations confer resistance to conventional chemotherapy, targeted therapy, blinatumomab, and chimeric antigen receptor T (CAR‐T) cell immunotherapy.^7^, ^8^, ^9^, ^10^, ^11^, ^12^ In addition, *IKZF1* plus (defined as *IKZF1* deletions that co‐occur with deletions in *CDKN2A*, *CDKN2B*, *PAX5*, or *PAR1* in the absence of an *ERG* deletion) is associated with worse outcomes in pediatric patients with B‐ALL^8^ and has been incorporated into the 2023 National Comprehensive Cancer Network (NCCN) guidelines. However, the prognostic implications of combining *IKZF1* aberrations and oncogenic activation in Ph‐like ALL have been marginally addressed. This study characterizes a novel high‐risk (HR) subgroup of Ph‐like ALL, which comprises a combination of *IKZF1* dysregulation and activated oncogenes related to Ph‐like ALL, which stratifies patients with Ph‐like ALL into higher‐ and lower‐risk categories. The new stratification may lead to innovative and effective targeted treatments for Ph‐like ALL.

## METHODS

### Study design

This multicenter, retrospective cohort study was designed to explore the clinical features of patients with Ph‐like ALL in the South China Hematology Consortium (SCHC). Patients with Ph‐like ALL were enrolled in five SCHC centers between September 2016 and December 2021. A cohort of patients with Ph^−^ ALL diagnosed at Nanfang Hospital (NFH) between January 2016 and December 2021 was introduced for comparison of survival. Informed consent was obtained from all the patients. This study was reviewed and approved by the Ethics Board of Nanfang Hospital, in accordance with the Declaration of Helsinki. The final follow‐up was conducted in September 2022. The diagnosis of Ph‐like ALL was generally based on the Chinese guidelines, NCCN guidelines, and World Health Organization (WHO) classification.^5^, ^6^, ^13^ The diagnosis criteria included ABL‐class fusions, *CRLF2* rearrangement or high expression, JAK‐STAT pathway alterations, Ras pathway mutations, and phosphorylated *(p)‐CRKL* or *p‐STAT5* high expression. Patients received chemotherapy or targeted drugs (TKIs and ruxolitinib) along with chemotherapy as frontline treatment. The chemotherapy regimens included Precision‐Classification‐Directed‐Target‐Total‐Therapy (PDT)‐ALL‐2016^11^, ^14^, ^15^ (for NFH) and the Chinese guideline for the treatment of adult ALL^5^ (for the other four SCHC centers). Allogeneic hematopoietic stem cell transplantation (allo‐HSCT) was recommended for patients who achieved complete remission (CR) if a donor was available. Salvage therapies, including intensive chemotherapy, targeted drugs, or immunotherapies (including blinatumomab and CAR‐T) were administrated to patients with minimal residual disease (MRD)‐positivity after induction and relapsed/refractory (R/R) disease.

### Statistical analysis

Categorical data was summarized by the proportion of patients. Quantitative data was described using median for central tendency and range or interquartile range (IQR) for distribution range. The *χ* ^2^ and Fisher's exact test were used to compare categorical variables. The *t*‐test and the Mann–Whitney *U* test were used to compare continuous variables. The Kaplan–Meier method was used to assess survival data using the log‐rank test. OS was estimated from the date of diagnosis to the date of last follow‐up or death from any cause. EFS was estimated from the date of diagnosis to the date of the last follow‐up or the first event (including nonresponse after induction, relapse, and death from any cause). To compare the prognosis of patients receiving allo‐HSCT or not, leukemia‐free survival (LFS) was estimated from the date of 6 months after diagnosis to the date of the last follow‐up, relapse, or death from any cause. The Cox proportional hazards regression model analyses including univariable and multivariable analyses were used to identify potential prognostic factors associated with OS and EFS. Statistical significance was defined as a two‐sided *p*‐value of <0.05. All statistical analyses were performed using R software (version 4.2.0, https://cran.r-project.org/).

## RESULTS

### Clinical characteristics

**Figure 1:**
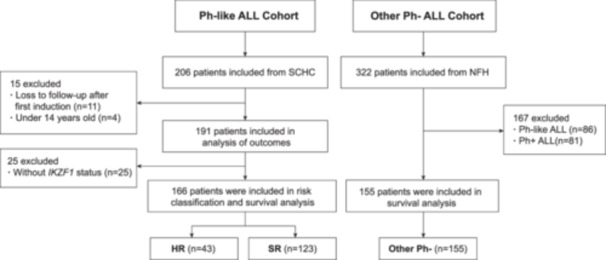
Study profile. HR, high‐risk; NFH, Nanfang Hospital; SCHC, South China Hematology Consortium; SR, standard‐risk.

A total of 191 adolescent and adult patients with Ph‐like ALL were included in the analysis (Figure 1), and their main clinical characteristics are summarized in Supporting Information S1: Table S1. The Ph‐like ALL cohort comprised 74 females and 117 males, ranging from 14 to 71 years, with a median age of 26 years (IQR 18–36) at diagnosis. The most common subtype was *CRLF2*, which was observed in 52 of 191 patients (27.2%) and included *CRLF2* rearrangement and *CRLF2* high expression. Patients with *EPOR*/*JAK2* rearrangement had higher WBC counts at diagnosis than those in other subgroups. A total of 68 patients (35.6%) received anti‐*CD19* and/or *CD22* CAR‐T therapy and 115 patients (60.2%) received allo‐HSCT. The other Ph^−^ ALL cohort included 155 patients with a median age of 28 years at diagnosis (range, 14–69 years). Among them, 97 (62.6%) underwent allo‐HSCT (Supporting Information S1: Table S1).

### Genomics overview and outcomes of the Ph‐like ALL cohort

**Figure 2:**
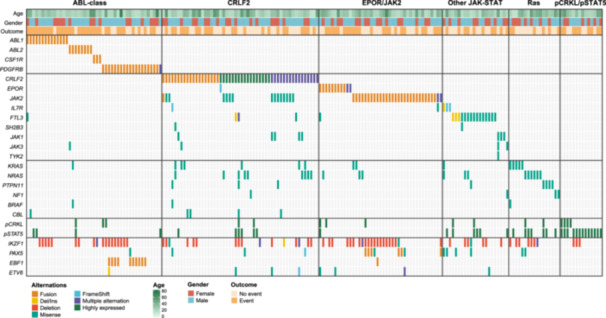
Genomic landscape of Philadelphia chromosome (Ph)‐like acute lymphoblastic leukemia (ALL). Data are presented for 191 patients with Ph‑like ALL, categorized based on specific genetic abnormalities. The categories include patients with ABL‑class fusions (*ABL1*, *ABL2*, *CSF1R*, and *PDGFRB*), *CRLF2* positive (rearrangement or high‐expression), *EPOR* or *JAK2* rearrangements, other JAK–STAT pathway mutations (*IL7R*, *FLT3*, *SH2B3*, *JAK1*, *JAK3*, and *TYK2*), Ras pathway mutations only (*KRAS*, *NRAS*, *PTPN11*, *NF1*, *BRAF*, and *CBL*), and *p‐CRKL/pSTAT5* high‐expression only. Alterations in transcription factors (*IKZF1*, *PAX5*, *EBF1*, and *ETV6*) are also shown. For specific details regarding fusion partner genes and mutations, please refer to Supporting Information S1: Table S3 and Supporting Information S1: Figure S2. *p‐CRKL*, phosphorylated *CRKL*; *p‐STAT5*, phosphorylated *STAT5*.

**Figure 3:**
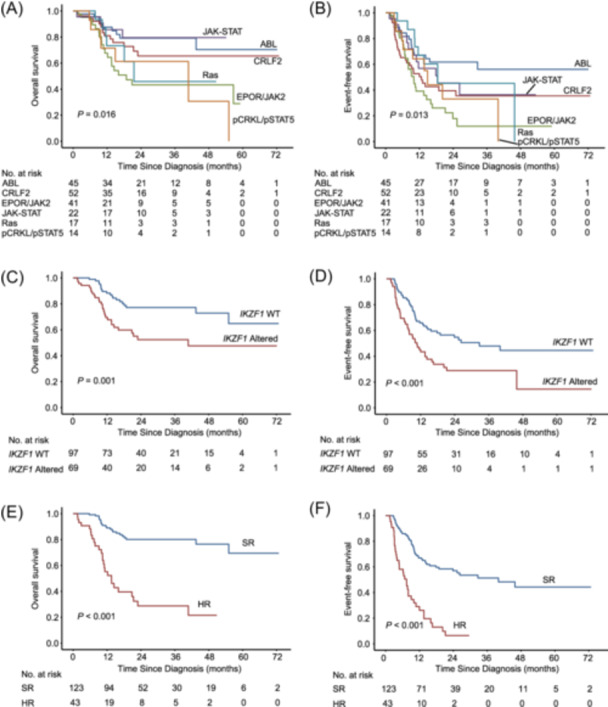
Overall survival (OS) and event‐free survival (EFS) of different subgroups in Philadelphia chromosome (Ph)‐like acute lymphoblastic leukemia (ALL). (A) OS and (B) EFS of 191 patients with different subtypes of Ph‐like ALL; (C) OS and (D) EFS of 166 patients stratified by *IKZF1* status; (E) OS and (F) EFS of 166 patients divided into HR and SR subgroups. HR, high‐risk; SR, standard‐risk.

Overall, activation of kinases or cytokine receptor signaling pathways was identified in 191 patients with Ph‐like ALL (Figure 2), including ABL‐class fusions (45/191, 23.6%; *ABL1*, *ABL2*, *CSF1R*, and *PDGFRB*), *CRLF2* positive (52/191, 27.2%; rearrangement or high‐expression), *EPOR* or *JAK2* rearrangement (41/191, 21.5%), JAK‐STAT pathway mutations (22/191, 11.5%; *IL7R*, *SH2B3*, *JAK1*, *JAK3*, and *FLT3*), Ras pathway mutations only (17/191, 8.9%; *KRAS*, *NRAS*, *PTPN11*, and *NF1*), *p‐CRKL* or *p‐STAT5* high‐expression only (14/191, 7.3%). The fusion partner genes and sequence mutations in genes of Janus and Ras kinase signaling pathways are summarized in Supporting Information S1: Table S3 and depicted in Supporting Information S1: Figure S1. Based on the available data, *IKZF1* alterations were detected in 42.2% (70/166) patients, including *IKZF1* deletions of exons 2–7, 2–8, 4–7, 4–8, and mutations. The median length of follow‐up was 24.3 months (range 3.2–72.8). Patients in subgroups of *EPOR*/*JAK2*‐rearrangement tended to have worse outcomes (median OS 18.8 months, median EFS 10.0 months), and patients in subgroups of ABL‐class fusions tended to have better outcomes in this study (both median OS and EFS were not reached) (Figure 3A,B). Because *IKZF1* alterations are associated with poor outcomes in B‐ALL, we investigated whether *IKZF1* alterations in Ph‐like ALL yielded similar results. Patients with *IKZF1* alterations had inferior outcomes compared to those without *IKZF1* alterations (3 y‐OS 50.6% and 74.7%, respectively, *p* = 0.001; 3 y‐EFS 27.7% and 56.6%, respectively, *p* < 0.001; Figure 3C,D). There were no significant differences in the proportions of treatments received between patients with and without *IKZF1* alterations (Supporting Information S1: Table S4).

### Activation of *CRLF2/EPOR/JAK2/p‐CRKL/p‐STAT5* plus inactivation of *IKZF1* exhibited the worst outcome

**Figure 4:**
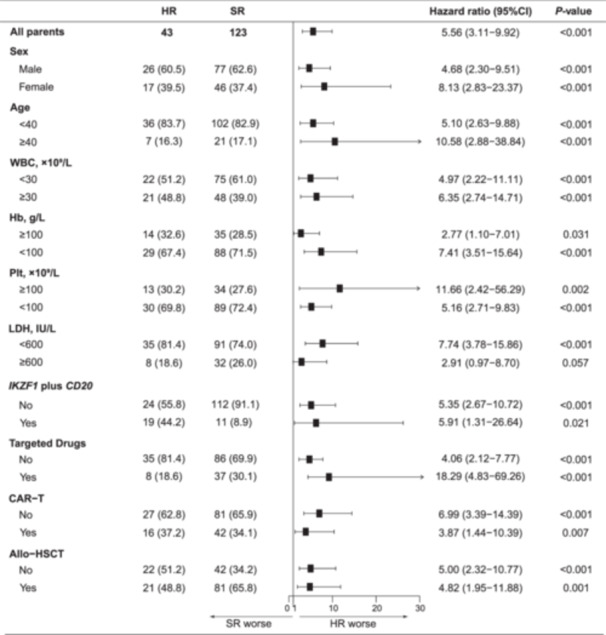
Subgroup analysis of overall survival (OS) in 166 patients. A total of 166 patients were divided into two groups, HR and SR, for subgroup analysis. Data are *n* (%). *IKZF1* plus *CD20* was defined as *IKZF1* deletions with *CD20*‐positive (≥20%). Targeted drugs included TKIs (imatinib, dasatinib, and orelabrutinib) and *JAK2* inhibitor (ruxolitinib). HR, high‐risk; SR, standard‐risk.

To identify the subgroup with the worst outcomes, we evaluated the combined effects of oncogenes activation and *IKZF1* inactivation in patients with Ph‐like ALL. A total of 166 patients with available *IKZF1* data were included in the subsequent analyses. To explore potential combinations associated with poor prognosis, we combined different molecular subtypes with *IKZF1* alterations to calculate the risk ratio for event occurrence (Supporting Information S1: Table S5). Considering that high *p‐CRKL* and *p‐STAT5* expression may occur simultaneously with other molecular abnormalities, the cases included in the analysis of *p‐CRKL/p‐STAT5* combined with *IKZF1* were those exhibiting *p‐CRKL* or *p‐STAT5* high expression, regardless of any co‐occurring molecular abnormalities. A statistically significant combination indicated a potentially HR subgroup in Ph‐like ALL, and all these combinations were merged into a new combination, namely *CRLF2/EPOR/JAK2/p‐CRKL/p‐STAT5* combined with *IKZF1* alterations. The new combination was classified as a HR subgroup, whereas the remaining subgroup was classified as a standard‐risk (SR) subgroup. Patients within the HR subgroup had worse prognoses than those within the standard‐risk subgroup (3 y‐OS 28.8% and 80.1%, respectively, and 2 y‐EFS 6.5% and 57.0%, respectively; Figure 3E,F). The median time from the first CR to relapse was 5 months (IQR: 1–11, HR) and 13 months (IQR: 4–25, SR), respectively. There were no significant differences in the proportions of treatments that patients received between HR and SR subgroups (Supporting Information S1: Table S6). Stratified subgroup analysis also revealed a similar trend of worse outcomes in HR than that in SR (Figure 4 and Supporting Information S1: Figure S3). Sensitivity analyses showed that dividing the cohort into NFH and the other four SCHC centers did not markedly affect the results (Supporting Information S1: Figure S4). Because the p‐*CRKL*/p‐*STAT5* subgroup was not strictly Ph‐like ALL, it was excluded and re‐analyzed. The combination of *CRLF2/EPOR/JAK2* and *IKZF1* still exhibited an unfavorable prognosis when excluding *p‐CRKL/p‐STAT5* (Supporting Information S1: Figure S5). Within the subgroup of *CRLF2/EPOR/JAK2*, *IKZF1* alterations were associated unfavorable prognosis (Supporting Information S1: Figure S5). Cox model analysis was performed to assess the prognostic factor for OS and EFS. There were statistically significant differences in outcomes between the HR subgroup and the SR subgroup (adjusted hazard ratio for OS = 4.55, 95% confidence interval [CI]: 2.35–8.81, *p* < 0.001, Table 1; adjusted hazard ratio for EFS = 3.27, 95% CI: 1.99–5.39, *p* < 0.001, Supporting Information S1: Table S2). In addition, patients within the HR subgroup exhibited adverse prognoses compared to the Ph^−^ ALL cohort, whereas SR and other Ph^−^ ALL showed comparable prognoses (Supporting Information S1: Figure S6). Finally, because the measurable residual disease (MRD, examined by flow cytometry with a sensitivity of 10^−4^) status has significant prognostic significance in the Cox model analysis, the prognostic effect of the HR feature in the context of treatment response was assessed. In patients with MRD‐positive after induction, outcomes were affected by the presence of HR feature, whereas outcomes of patients with MRD‐negative after induction were not significantly affected by HR feature (Supporting Information S1: Figure S7).

**Table 1:** Univariate analysis and multivariate analysis for OS (*n* = 166).

Variable		Univariate analysis for OS		Multivariate analysis for OS	
		Unadjusted hazard ratio (95% CI)	*p*	Adjusted hazard ratio (95% CI)	*p*
Age, years	≥40 versus <40	1.76 (0.90–3.48)	0.101	2.11 (1.00–4.46)	0.051
WBC count, ×10^9^/L	≥30 versus <30	1.54 (0.88–2.72)	0.134	1.76 (0.95–3.24)	0.071
*IKZF1* plus *CD20*	Yes versus No	2.15 (1.15–4.02)	**0.016**	1.36 (0.66–2.79)	0.399
MRD after induction	Neg versus Pos	0.44 (0.22–0.86)	**0.016**	0.42 (0.20–0.88)	**0.022**
Targeted drugs	Yes versus No	0.64 (0.32–1.29)	0.212	0.95 (0.46–1.97)	0.895
CAR‐T	Yes versus No	1.03 (0.63–1.68)	0.914	0.70 (0.37–1.32)	0.270
Allo‐HSCT	Yes versus No	0.38 (0.25–0.59)	**<0.001**	0.26 (0.14–0.49)	**<0.001**
Risk stratification	HR versus SR	5.56 (3.11–9.92)	**<0.001**	4.55 (2.35–8.81)	**<0.001**

*Note*: MRD negative was defined as less than 1 × 10^−4^, and MRD positive was defined as 1 × 10^−4^ or higher. Targeted drugs including TKIs (imatinib, dasatinib, and orelabrutinib), JAK2 inhibitor (ruxolitinib). A total of 45 (27.1%) patients received targeted drugs. The bold values indicate statistical significant at *p* < 0.05.

Abbreviations: Allo‐HSCT, allogeneic stem cell transplantation; CAR‐T, chimeric antigen receptor T; CI, confidence interval; HR, high‐risk; MRD, measurable residual disease; Neg, negative; OS, overall survival; Pos, positive; SR, standard‐risk; WBC, white blood cell.

### Immunotherapies and allo‐HSCT in patients within the HR subgroup of Ph‐like ALL

**Figure 5:**
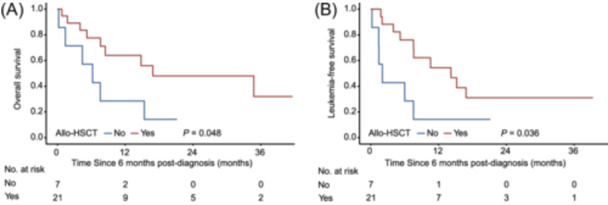
Overall survival (OS) and leukemia‐free survival (LFS) of patients within HR subgroup according to allogeneic hematopoietic stem cell transplantation (allo‐HSCT) (*n* = 26). (A) OS and LFS (B) of 26 HR patients based on whether they received allo‐HSCT. HR, high‐risk.

Then we focused on evaluating the effectiveness of immunotherapies and allo‐HSCT in improving the prognosis of patients within the HR subgroup. Among the 43 patients exhibiting HR feature, four (9.3%) received blinatumomab. Of these, two underwent treatment due to induction failure, one with CR received treatment post‐induction to improve prognosis, while one was treated following relapse after transplantation. Both patients who received blinatumomab due to induction failure died of disease progression, whereas the other two patients remained alive. Additionally, 16 (37.2%) of the 43 HR patients underwent CAR‐T therapy, with a median time from diagnosis to CAR‐T administration recorded at 5 months (IQR: 3–15). Notably, all 16 patients achieved CR post‐CAR‐T administration, but eight experienced relapses, with a median time to relapse of 4 months (range 2–16). Furthermore, 21 (48.8%) of the 43 HR patients underwent allo‐HSCT, with a median time from diagnosis to allo‐HSCT of 6 months (IQR: 5–7), comprising 20 at CR1 and one at CR2. Nine of the 21 (42.9%) patients received CAR‐T prior to transplantation. A landmark analysis, which included only patients alive and in remission at 6 months after diagnosis, was conducted to evaluate post‐allo‐HSCT outcomes. A total of 26 patients were included in this analysis. Undergoing allo‐HSCT was associated with better LFS and OS (Figure 5).

## DISCUSSION

Ph‐like ALL is characterized and classified based on the activation of multiple oncogenes, such as *ABL1*, *CRLF2*, and *EPOR* rearrangements, leading to a heterogeneous disease phenotype. Meanwhile, Ph‐like ALL shares a high frequency of TSG *IKZF1* deletions with the Ph‐positive subset. *IKZF1* aberration is considered as a prognostic factor in B‐ALL, particularly in Ph‐positive and Ph‐like ALL, leading to adverse outcomes.^2^, ^16^, ^17^ In Ph‐positive ALL, *IKZF1* deletions contribute to leukemogenesis and TKIs resistance in conjunction with *BCR::ABL1* fusion.^18^, ^19^ In pediatric B‐ALL, the AIEOP‐BFM group defined *IKZF1plus* as *IKZF1* deletions combined with *CDKN2A*, *CDKN2B*, *PAX5*, or *PAR1* deletions without an *ERG* deletion, which confers the worst outcomes.^8^ Deletion of 22q11.22 is also common in pediatric Ph‐like ALL, and the combination of 22q11.22 and *IKZF1* deletions can refine the risk stratification.^20^ In adult B‐ALL, our previous study demonstrated that *IKZF1* deletions plus *CD20* positivity correlated with a very poor prognostic profile.^11^ These findings characterize the subgroups with poor prognoses in B‐ALL and refine the *IKZF1*‐based prognostic stratification. In the current study, we revealed a synergistic effect of *CRLF2/EPOR/JAK2/p‐CRKL/p‐STAT5* and *IKZF1* in Ph‐like ALL, which resulted in poor outcomes and shaped the Ph‐like classification into HR and SR subgroups.

The current classification of Ph‐like ALL is inadequate for predicting patient outcomes. The re‐classification of HR and SR subgroups enabled the identification of patients with poor prognoses, which could be valuable for risk classification and seeking more effective targeted treatments. Although gene expression profiling analysis is the standard method for diagnosing Ph‐like ALL,^1^, ^21^ it remains crucial to develop more effective diagnostic approaches. We employed a comprehensive screening strategy involving flow cytometry, fluorescence in situ hybridization, reverse‐transcription polymerase chain reaction, targeted next‐generation sequencing, RNA sequencing, and whole‐exome sequencing. Furthermore, the challenge of treatment lies in bridging the gap between the identification of novel subsets and the lagging development of targeted strategies. Optimal therapeutic approaches for Ph‐like ALL have not yet been clearly characterized. ABL‐class Ph‐like ALL is sensitive to TKIs,^2^ and our results showed that this subgroup had a relatively favorable prognosis in Ph‐like ALL. *JAK2* inhibitor ruxolitinib showed promising results but with high doses. Other targeted drugs, such as Ras and MEK inhibitors in the Ras class, remain far from bench to bedside.^22^ In addition, immunotherapy bi‐specific *CD3*/*CD19* antibodies blinatumomab has shown promising results in R/R and *CRLF2*‐rearranged Ph‐like ALL.^23^, ^24^ However, resistance remains a challenge, especially in patients with *IKZF1* deletions and *IKZF1* plus.^9^ It is worth mentioning that combining Hyper‐CVAD and blinatumomab in the frontline setting has led to improved outcomes, with a 3‐year OS rate of 76% in patients with high‐risk features (including *CRLF2* and *TP53*).^25^ As for CAR‐T therapy, a significant portion of patients (42.9%) received CAR‐T prior to transplantation. Therefore, the role of CAR‐T therapy in HR subgroup cannot be dismissed completely, warranting further investigation. Finally, allo‐HSCT remains crucial for achieving favorable survival outcomes for Ph‐like ALL.^26^, ^27^ In this study, allo‐HSCT was associated with improved outcomes in patients within the HR subgroup of Ph‐like ALL. However, given that the small numbers of patients (*n* = 26) included in the analysis, these findings should be interpreted with caution.

Although transplantation may improve the prognosis of patients with HR Ph‐like ALL to some extent, their prognosis remains worse compared to the SR subgroup. This disparity may be attributed to the lack of targeted therapies for oncogenes like *CRLF2*, *EPOR*, and *JAK2*, particularly for inactivated TSG *IKZF1*. *IKZF1* alterations will lead to acquisition of stem cell‐like features, metabolic reprogramming, and resistance to drugs and immunotherapies,^7^, ^9^, ^10^, ^16^, ^17^, ^28^, ^29^, ^30^ underscoring the importance of targeting *IKZF1* deletions in Ph‐like ALL. In fact, there is still a lack of knowledge regarding how to target *IKZF1* deletion ALL. We previously reported that tucidinostat targets *IKZF1* deletions ALL by restoring *IKZF1* expression in vitro and in vivo.^10^ In the current study, we identified a novel subgroup with poor outcomes characterized by *IKZF1* alterations combined with *CRLF2/EPOR/JAK2* rearrangements or *p‐CRKL/p‐STAT5* high expression. This suggests the necessity of a dual‐targeting approach for *IKZF1* and oncogenes like *CRLF2*, *EPOR*, and *JAK2* in the HR subset, and further translational research on underlying mechanisms is necessary.

This study has several limitations. First, the definition of the HR subgroup included *p‐CRKL/p‐STAT5* only measured in NFH, which might limit the generalizability of the findings. Second, previous studies suggested that patients with *CRLF2* overexpression have worse outcomes,^31^ whereas our study found noninferior outcomes in patients with *CRLF2* high expression. This inconsistency may be attributed to variations in the criteria for defining *CRLF2* high expression, differences in the frequency of *IKZF1* alterations or *JAK2* mutations, and potential racial and ethnic disparities. Third, *IKZF1* status data were not available for 25 of the 191 patients. These cases were excluded from the analysis of HR and SR classification, which could have introduced a potential bias. Moreover, due to a lack of systematic assessment of abnormalities of *IKZF1* plus,^8^ this study did not include *IKZF1* plus, which may be relevant to the adverse outcomes of patients within the HR subgroup. Finally, as an observational study, there is the risk of unmeasured confounding factors that may counteract the apparent contribution of the HR feature as an independent risk factor for Ph‐like ALL.

In conclusion, our study identified a clinically distinct HR subgroup that serves as a valuable prognostic feature of Ph‐like ALL and may provide guidance for refining Ph‐like ALL risk stratification for cutting‐edge therapeutic approaches.

## AUTHOR CONTRIBUTIONS

Hongsheng Zhou and Zicong Huang contributed to the conception of the study. Bingqing Tang, Kangyu Huang, Xin Li, Weihua Zhao, Yang Xu, and Li Xuan contributed to the provision of study materials and acquisition of the clinical data. Zicong Huang, Jia Li, Shiyu Deng, and Zihong Cai performed the statistical analyses. Zicong Huang, Ling Zhang, and Xiaoyuan Gong drafted the manuscript. Hongsheng Zhou, Suning Chen, Ying Wang, and Qifa Liu revised the final manuscript. All authors reviewed the final manuscript and consented to submission. Zicong Huang, Ling Zhang, Xiaoyuan Gong, and Jia Li contributed equally to this work.

## CONFLICT OF INTEREST STATEMENT

Hongsheng Zhou reports research grants from CHIPSCREEN and JW Therapeutics. The other authors declare no conflict of interest.

## FUNDING

This study was supported by the National Natural Science Foundation of China (NSFC, 82170163, 81970147, to Hongsheng Zhou), the National Key Research and Development Program of China (2022YFC2502600‐5, to Li Xuan), CHIPSCREEN (to Hongsheng Zhou), and JW Therapeutics (to Hongsheng Zhou).

## Supporting information

Supplementary information.

## Data Availability

The data that support the findings of this study are available from the corresponding author upon reasonable request.

## References

[1] Den Boer ML , van Slegtenhorst M , De Menezes RX , et al. A subtype of childhood acute lymphoblastic leukaemia with poor treatment outcome: a genome‐wide classification study. Lancet Oncol. 2009;10(2):125‐134. 10.1016/S1470-2045(08)70339-5 19138562 PMC2707020

[2] Roberts KG , Li Y , Payne‐Turner D , et al. Targetable kinase‐activating lesions in Ph‐like acute lymphoblastic leukemia. N Engl J Med. 2014;371(11):1005‐1015. 10.1056/NEJMoa1403088 25207766 PMC4191900

[3] Roberts KG , Gu Z , Payne‐Turner D , et al. High frequency and poor outcome of Philadelphia chromosome–like acute lymphoblastic leukemia in adults. J Clin Oncol. 2017;35(4):394‐401. 10.1200/JCO.2016.69.0073 27870571 PMC5455698

[4] Moorman AV , Barretta E , Butler ER , et al. Prognostic impact of chromosomal abnormalities and copy number alterations in adult B‐cell precursor acute lymphoblastic leukaemia: a UKALL14 study. Leukemia. 2022;36(3):625‐636. 10.1038/s41375-021-01448-2 34657128 PMC8885405

[5] Hematology Oncology Committee, Chinese Anti‐Cancer Association, Leukemia & Lymphoma Group, Chinese Society of Hematology, Chinese Medical Association . Chinese guidelines for diagnosis and treatment of adult acute lymphoblastic leukemia [In Chinese]. Zhonghua Xue Ye Xue Za Zhi. 2021;42(9):705‐716. 10.3760/cma.j.issn.0253-2727.2021.09.001 34753224 PMC8607046

[6] Brown PA , Shah B , Advani A , et al. Acute lymphoblastic leukemia, version 2.2021, NCCN Clinical Practice Guidelines in Oncology. J Natl Compr Cancer Netw. 2021;19(9):1079‐1109. 10.6004/jnccn.2021.0042 34551384

[7] Mullighan CG , Su X , Zhang J , et al. Deletion of *IKZF1* and prognosis in acute lymphoblastic leukemia. N Engl J Med. 2009;360(5):470‐480. 10.1056/NEJMoa0808253 19129520 PMC2674612

[8] Stanulla M , Dagdan E , Zaliova M , et al. *IKZF1* ^plus^ defines a new minimal residual disease–dependent very‐poor prognostic profile in pediatric B‐cell precursor acute lymphoblastic leukemia. J Clin Oncol. 2018;36(12):1240‐1249. 10.1200/JCO.2017.74.3617 29498923

[9] Foà R , Bassan R , Vitale A , et al. Dasatinib–blinatumomab for Ph‐positive acute lymphoblastic leukemia in adults. N Engl J Med. 2020;383(17):1613‐1623. 10.1056/NEJMoa2016272 33085860

[10] Huang K , Tang B , Cai Z , et al. HDACi targets IKZF1 deletion high‐risk acute lymphoblastic leukemia by inducing IKZF1 expression and rescuing IKZF1 function in vitro and in vivo. Blood. 2021;138(Suppl 1):514. 10.1182/blood-2021-152926

[11] Tang B , Cai Z , Wang Z , et al. Allogeneic hematopoietic stem cell transplantation overcome the poor prognosis of patients with IKZF1plus CD20–a very high‐risk subtype in B‐cell acute lymphoblastic leukemia. Bone Marrow Transpl. 2022;57(12):1751‐1757. 10.1038/s41409-022-01797-1 36056210

[12] Leahy AB , Devine KJ , Li Y , et al. Impact of high‐risk cytogenetics on outcomes for children and young adults receiving CD19‐directed CAR T‐cell therapy. Blood. 2022;139(14):2173‐2185. 10.1182/blood.2021012727 34871373 PMC8990372

[13] Swerdlow SH , Campo E , Harris NL . WHO Classification of Tumours of Haematopoietic and Lymphoid Tissues. International Agency for Research on Cancer; 2017.

[14] Zhang H , Fan Z , Huang F , et al. Busulfan plus cyclophosphamide versus total body irradiation plus cyclophosphamide for adults acute B lymphoblastic leukemia: an open‐label, multicenter, phase III trial. J Clin Oncol. 2023;41(2):343‐353. 10.1200/JCO.22.00767 36084276 PMC9839269

[15] Cai Z , Liu Y , Tang B , et al. Dynamics of minimal residual disease defines a novel risk‐classification and the role of allo‐HSCT in adult Ph‐negative B‐cell acute lymphoblastic leukemia. Leuk Lymphoma. 2022;63(13):3181‐3190. 10.1080/10428194.2022.2115841 36098226

[16] Georgopoulos K . Acute lymphoblastic leukemia—on the wings of IKAROS. N Engl J Med. 2009;360(5):524‐526. 10.1056/NEJMe0809819 19131438

[17] Martinelli G , Iacobucci I , Storlazzi CT , et al. *IKZF1* (Ikaros) deletions in *BCR‐ABL1*–positive acute lymphoblastic leukemia are associated with short disease‐free survival and high rate of cumulative incidence of relapse: a GIMEMA AL WP report. J Clin Oncol. 2009;27(31):5202‐5207. 10.1200/JCO.2008.21.6408 19770381

[18] van der Veer A , Zaliova M , Mottadelli F , et al. IKZF1 status as a prognostic feature in BCR‐ABL1–positive childhood ALL. Blood. 2014;123(11):1691‐1698. 10.1182/blood-2013-06-509794 24366361

[19] Churchman ML , Low J , Qu C , et al. Efficacy of retinoids in IKZF1‐mutated BCR‐ABL1 acute lymphoblastic leukemia. Cancer Cell. 2015;28(3):343‐356. 10.1016/j.ccell.2015.07.016 26321221 PMC4573904

[20] Mangum DS , Meyer JA , Mason CC , et al. Association of combined focal 22q11.22 deletion and *IKZF1* alterations with outcomes in childhood acute lymphoblastic leukemia. JAMA Oncol. 2021;7(10):1521. 10.1001/jamaoncol.2021.2723 34410295 PMC8377604

[21] Harvey RC , Kang H , Roberts KG , et al. Development and validation of a highly sensitive and specific gene expression classifier to prospectively screen and identify B‐precursor acute lymphoblastic leukemia (ALL) patients with a Philadelphia chromosome‐like (“Ph‐like” or “BCR‐ABL1‐Like”) signature for therapeutic targeting and clinical intervention. Blood. 2013;122(21):826. 10.1182/blood.V122.21.826.826

[22] Sasaki K , Yamauchi T , Semba Y , et al. Genome‐wide CRISPR‐Cas9 screen identifies rationally designed combination therapies for *CRLF2‐*rearranged Ph‐like ALL. Blood. 2022;139(5):748‐760. 10.1182/blood.2021012976 34587248 PMC9632759

[23] Zhao Y , Aldoss I , Qu C , et al. Tumor‐intrinsic and ‐extrinsic determinants of response to blinatumomab in adults with B‐ALL. Blood. 2021;137(4):471‐484. 10.1182/blood.2020006287 32881995 PMC7845009

[24] Jabbour EJ , Short NJ , Jain N , et al. Blinatumomab is associated with favorable outcomes in patients with B‐cell lineage acute lymphoblastic leukemia and positive measurable residual disease at a threshold of 10^−4^ and higher. Am J Hematol. 2022;97(9):1135‐1141. 10.1002/ajh.26634 35713551

[25] Jabbour E , Short NJ , Jain N , et al. Hyper‐CVAD and sequential blinatumomab for newly diagnosed Philadelphia chromosome‐negative B‐cell acute lymphocytic leukaemia: a single‐arm, single‐centre, phase 2 trial. Lancet Haematol. 2022;9(12):e878‐e885. 10.1016/S2352-3026(22)00285-X 36279879

[26] Cho H , Kim Y , Yoon JH , et al. Non‐inferior long‐term outcomes of adults with Philadelphia chromosome‐like acute lymphoblastic leukemia. Bone Marrow Transpl. 2021;56(8):1953‐1963. 10.1038/s41409-021-01253-6 PMC8338554 33824439

[27] Aldoss I , Yang D , Tomasian V , et al. Outcomes of allogeneic hematopoietic cell transplantation in adults with fusions associated with Ph‐like ALL. Blood Adv. 2022;6(17):4936‐4948. 10.1182/bloodadvances.2022007597 35816633 PMC9631622

[28] Georgopoulos K , Bigby M , Wang JH , et al. The ikaros gene is required for the development of all lymphoid lineages. Cell. 1994;79(1):143‐156. 10.1016/0092-8674(94)90407-3 7923373

[29] Joshi I , Yoshida T , Jena N , et al. Loss of Ikaros DNA‐binding function confers integrin‐dependent survival on pre‐B cells and progression to acute lymphoblastic leukemia. Nat Immunol. 2014;15(3):294‐304. 10.1038/ni.2821 24509510 PMC4494688

[30] Chan LN , Chen Z , Braas D , et al. Metabolic gatekeeper function of B‐lymphoid transcription factors. Nature. 2017;542(7642):479‐483. 10.1038/nature21076 28192788 PMC5621518

[31] Harvey RC , Mullighan CG , Chen IM , et al. Rearrangement of CRLF2 is associated with mutation of JAK kinases, alteration of IKZF1, Hispanic/Latino ethnicity, and a poor outcome in pediatric B‐progenitor acute lymphoblastic leukemia. Blood. 2010;115(26):5312‐5321. 10.1182/blood-2009-09-245944 20139093 PMC2902132

