# Supplementary material for: An integrated classification of tumor suppressor IKZF1 inactivation and oncogenic activation in Philadelphia chromosome‐like acute lymphoblastic leukemia
Source: Hemasphere. 2024 May 21;8(5):e82. doi: 10.1002/hem3.82 (PMC11106797; doi:10.1002/hem3.82)
Supplement: Supplementary file 1 — Supplementary information. [file HEM3-8-e82-s001.pdf]

Supplemental Data

for

An Integrated Classification of Tumor Suppressor *IKZF1* Inactivation and Oncogenic Activation in Philadelphia Chromosome-like Acute Lymphoblastic Leukemia

Zicong Huang, Ling Zhang, Xiaoyuan Gong, Jia Li, Shiyu Deng, Zihong Cai, Bingqing Tang, Kangyu Huang, Xin Li, Weihua Zhao, Yang Xu, Li Xuan, Qifa Liu, Ying Wang, Suning Chen, Hongsheng Zhou

TABLE OF CONTENTS

Supplemental Methods 2

Supplemental Figures.. 5

Supplemental Tables.. 13

Supplementary Methods

Detection of *IKZF1* deletion

Genomic DNA from bone marrow cell smears or fresh bone marrow samples were extracted using the DNeasy Blood & Tissue Kit (QIAGEN, Germany) according to the manufacturer's protocols. *IKZF1* deletion was detected by breakpoint-specific multiplex fluorescent PCR as previously described (exons deletions of 2-7, 2-8, 4-7, 4-8) [1]. Because of the lack of DNA samples, the *IKZF1* status of 7 samples were detected using RNA-seq data according to Brown et al. described previously[2]. *IKZF1* deletion transcripts (del2-7, del2-8, del4-7, and del4-8) contribute 5% or more of the total TPM of *IKZF1* are considered *IKZF1* deletion.

Measurement of *CRLF2, p-CRKL* and *p-STAT5*

Flow cytometric immunophenotyping was assessed via 8-color multiparameter flow cytometry (FACSCanto II, BD Biosciences) and the data was analyzed using Diva (BD Biosciences). In general, after excluding debris and select the single-cell population, the *CD45*/side scatter (SSC) plot was typically utilized to establish a gate for identifying abnormal cells, which were characterized by *CD45dim* and intermediate SSC values. Abnormal cells were further defined as the B lineage population (*CD19*+ *cCD79a*+, *CD10*+, *CD34*+, *CD13*+, *CD33*+), while excluding cells positive for *cMPO, cCD3, CD64*, and *CD117*, and the following analysis was based on abnormal cell populations. Antibodies to *CRLF2* (BD Biosciences, #563340), *p-CRKL* (BD Biosciences, #560789) and *p-STAT5* (BD Biosciences, #560311) were added to screen Ph-like ALL as Roberts et al. reported previously[3]. Isotype controls were employed to set gates to distinguish negative and positive populations based on the fluorescence intensity. The high expression of *CRLF2/p-CRKL/p-STAT5* was defined as more than 20% of cells expressing *CRLF2/p-CRKL/p-STAT5*. Measurable residual disease (MRD) was assessed via 8-color multiparameter flow cytometry using

either leukemia-associated immunophenotype (LAIPs) or difference from normal assessment with a sensitivity of 1×10^{-4} cells. MRD negative was defined as less than 1×10^{-4}, and MRD positive was defined as 1×10^{-4} or higher.

Fluorescence in situ hybridization (FISH)

FISH probes were designed for A*BL1, ABL2, JAK2, EPOR, CRLF2, CSF1R, PDGFRB*, and *P2RY8* gene breakpoints as Roberts et al. reported previously[3], and were used to screen Ph-like ALL. FISH probes for *BCR*, *ABL1*, *ETV6*, *RUNX1*, *MLL*, and *E2A*, were used to screen patients carrying *BCR*::*ABL1*, *ETV6*::*RUNX1*, *MLL* and *E2A* rearrangement.

Targeted next-generation sequencing (NGS)

Gene mutational status was detected by NGS with a panel of 167 targets using mononuclear cell DNA extracted from bone marrow specimens at diagnosis. DNA extraction, sequencing library preparation, and targeted sequencing were performed following the methods as previously described[4].

Missing values processing

To include data with missing values for Cox regression analysis, we imputed missing data by multiple imputations using the mice of R package (Version 3.14.0) with a predictive mean matching method using default parameters (maxit = 5). Cases with missing WBC count (1/191, 0.05%), hemoglobin (1/191, 0.05%), platelet count (1/191, 0.05%), and LDH (18/191, 9.42%) included embedded values (Supplemental Figure 6).

References

1. Caye, A. *et al.* Breakpoint-specific multiplex polymerase chain reaction allows the detection of IKZF1 intragenic deletions and minimal residual disease monitoring in B-cell precursor acute lymphoblastic leukemia. *Haematologica* **98**, 597–601 (2013).
2. Brown, L. M. *et al.* The application of RNA sequencing for the diagnosis and genomic classification of pediatric acute lymphoblastic leukemia. *Blood Adv.* **4**, 930–942 (2020).

3. Roberts, K. G. *et al.* Targetable Kinase-Activating Lesions in Ph-like Acute Lymphoblastic Leukemia. *N. Engl. J. Med.* **371**, 1005–1015 (2014).
4. Tang, B. *et al.* Allogeneic hematopoietic stem cell transplantation overcome the poor prognosis of patients with IKZF1plus CD20–a very high-risk subtype in B-cell acute lymphoblastic leukemia. *Bone Marrow Transplant.* (2022) doi:10.1038/s41409-022-01797-1.

Supplemental Figure 1. Flow chart for identification of Ph-like ALL

Abbreviations: FISH, fluorescence in situ hybridization; RT-PCR, reverse transcription-polymerase chain reaction; *p-CRKL*, phosphorylated *CRKL*; *p-STAT5*, phosphorylated *STAT5; NGS, next-generation sequencing; WES, whole-exome sequencing*

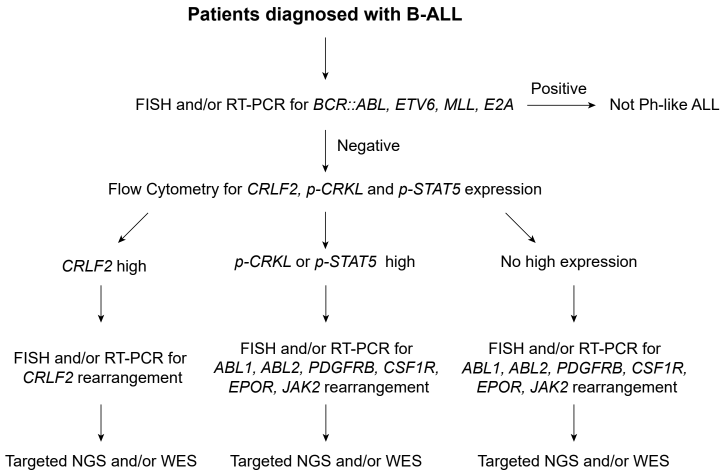

Supplemental Figure 2. Janus and Ras kinase mutations

(A) Protein plot of sequence mutations in *JAK1* and *JAK2*. (B) Protein plot of sequence mutations in Ras pathway. Abbreviations: FERM, band 4.1 ezrin, radixin, and moesin domain; SH2, Src homology 2 domain.

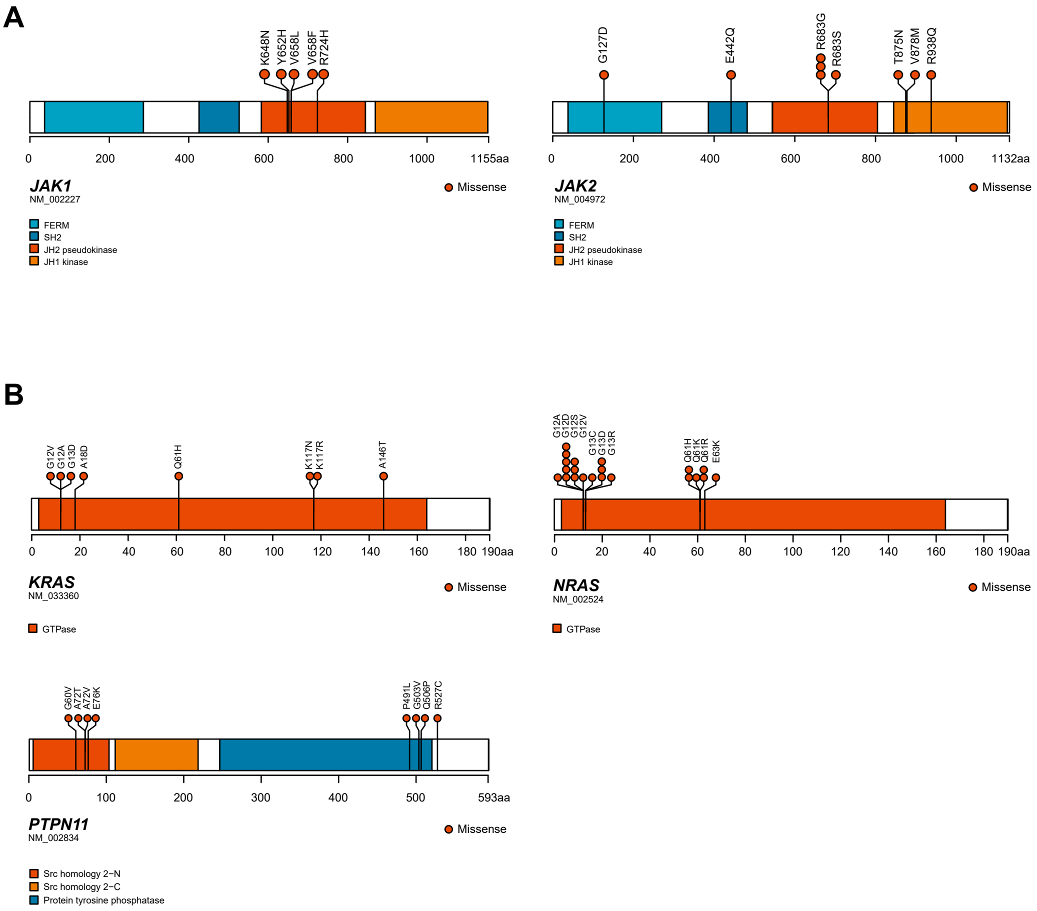

Supplemental Figure 3. Subgroup analysis of EFS in 166 patients

A total of 166 patients were divided into two groups, HR and SR, for subgroup analysis. Data are n (%). *IKZF1* plus *CD20* was defined as *IKZF1* deletions with *CD20*-positive (≥ 20%). Targeted drugs included TKIs (imatinib, dasatinib and orelabrutinib) and *JAK2* inhibitor (ruxolitinib).

Abbreviations: HR, high-risk; SR, standard-risk.

	HR	SR	Hazard ratio (95%CI)	*P*-value
**All parents**	**43**	**123**	4.04 (2.60−6.27)	<0.001
**Sex**				
Male	26 (60.47)	77 (62.60)	4.85 (2.80−8.40)	<0.001
Female	17 (39.53)	46 (37.40)	3.00 (1.43−6.30)	0.004
**Age**				
<40	36 (83.72)	102 (82.93)	3.65 (2.25−5.92)	<0.001
≥40	7 (16.28)	21 (17.07)	6.76 (2.22−20.59)	0.001
**WBC, ×10⁹/L**				
<30	22 (51.16)	75 (60.98)	3.67 (2.04−6.63)	<0.001
≥30	21 (48.84)	48 (39.02)	4.63 (2.37−9.06)	<0.001
**Hb, g/L**				
≥100	14 (32.56)	35 (28.46)	4.61 (2.15−9.89)	<0.001
<100	29 (67.44)	88 (71.54)	3.76 (2.19−6.45)	<0.001
**Plt, ×10⁹/L**				
≥100	13 (30.23)	34 (27.64)	4.49 (1.91−10.55)	0.001
<100	30 (69.77)	89 (72.36)	3.91 (2.33−6.57)	<0.001
**LDH, IU/L**				
<600	35 (81.40)	91 (73.98)	5.26 (3.12−8.85)	<0.001
≥600	8 (18.60)	32 (26.02)	2.15 (0.84−5.51)	0.111
IKZF1* plus *CD20				
No	24 (55.81)	112 (91.06)	3.82 (2.21−6.61)	<0.001
Yes	19 (44.19)	11 (8.94)	3.23 (1.25−8.36)	0.016
**Targeted Drugs**				
No	35 (81.40)	86 (69.92)	3.54 (2.13−5.88)	<0.001
Yes	8 (18.60)	37 (30.08)	7.00 (2.70−18.17)	<0.001
**CAR−T**				
No	27 (62.79)	81 (65.85)	4.76 (2.63−8.60)	<0.001
Yes	16 (37.21)	42 (34.15)	3.15 (1.62−6.10)	0.001
**Allo−HSCT**				
No	22 (51.16)	42 (34.15)	3.42 (1.81−6.45)	<0.001
Yes	21 (48.84)	81 (65.85)	4.12 (2.23−7.63)	<0.001

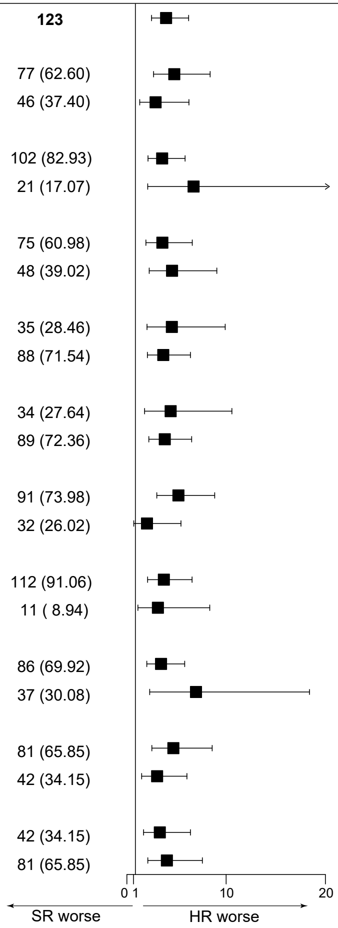

Supplemental Figure 4. OS and EFS of 166 patients in single cohort

(A) OS of 75 patients from NFH. (B) OS of 91 patients from other four centers of SCHC. (C) EFS of 75 patients from NFH. (D) OS of 91 patients from other four centers of SCHC.

Abbreviations: NFH, Nanfang Hospital; SCHC, South China Hematology Consortium; HR, high-risk; SR, standard-risk.

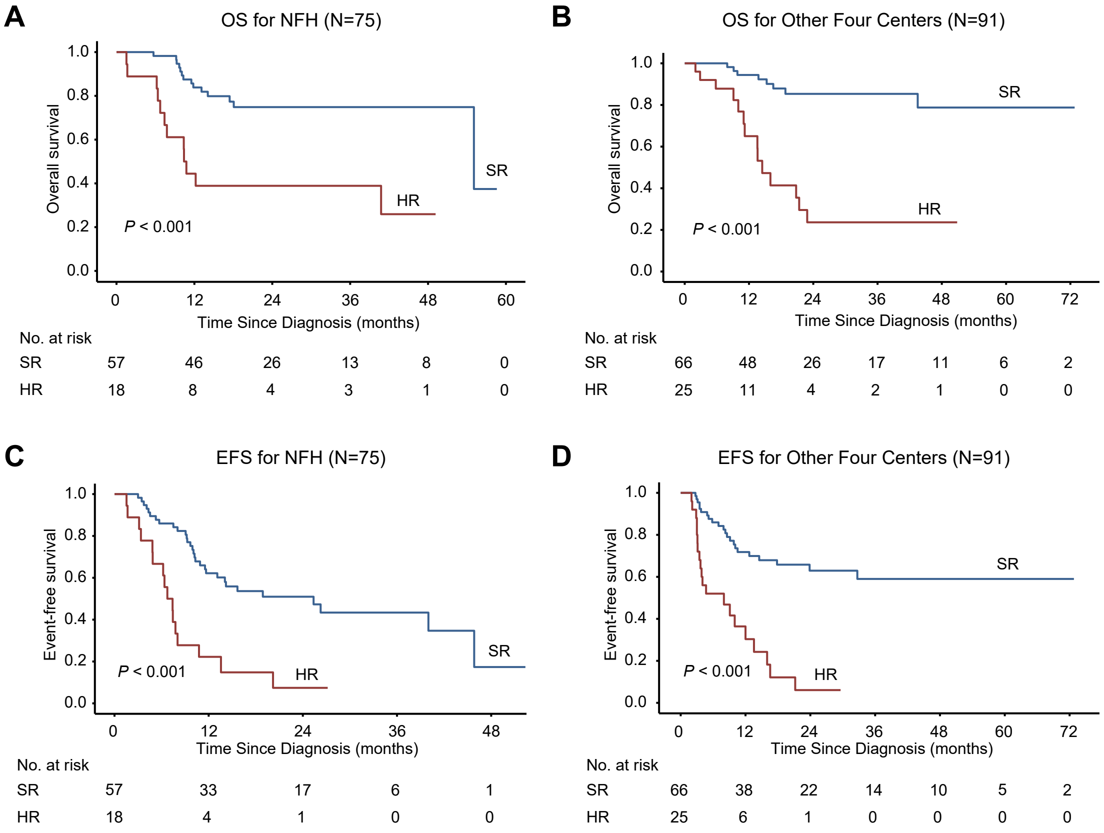

Supplemental Figure 5. OS and EFS of patients grouped by *CRLF2/EPOR/JAK2* plus *IKZF1*

(A) OS and (B) EFS of patients with or without *CRLF2/EPOR/JAK2* plus *IKZF1*.

(C) OS and (D) EFS of patients with or without *IKZF1* alterations within the *CRLF2/EPOR/JAK2* subgroup.

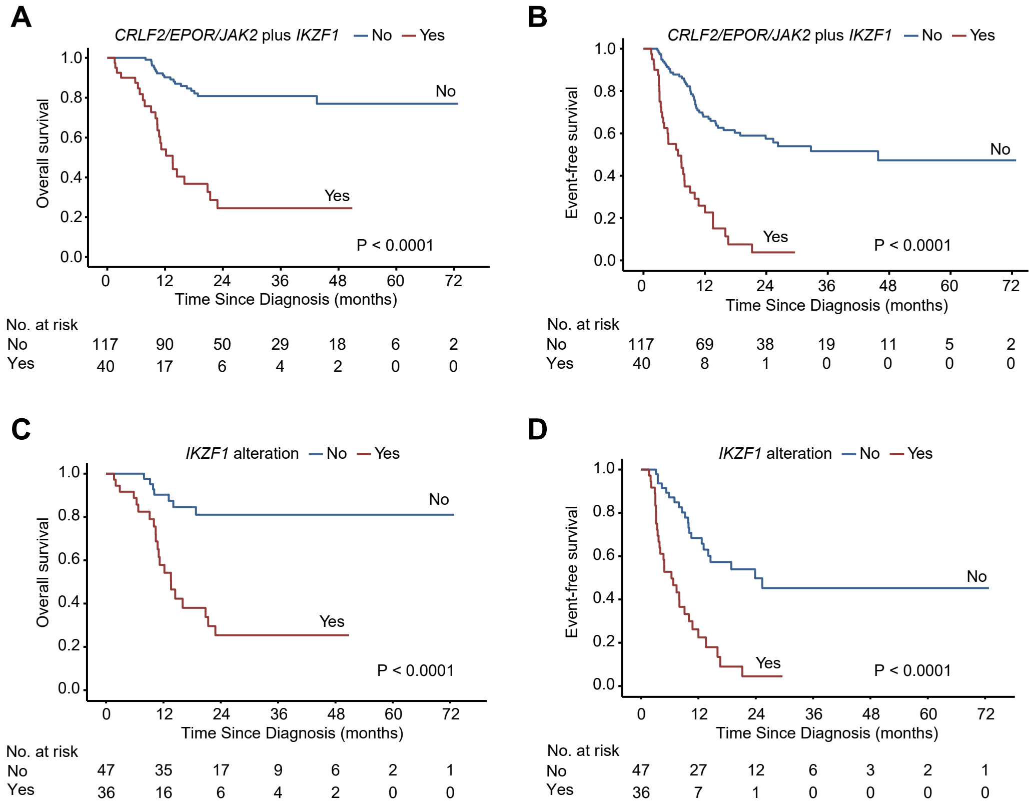

Supplemental Figure 6. OS and EFS of patients within HR and SR subgroups compared to Other Ph- cohort

(A) OS and (B) EFS of patients stratified by HR, SR and Other Ph- ALL.

Abbreviations: HR, high-risk; SR, standard-risk.

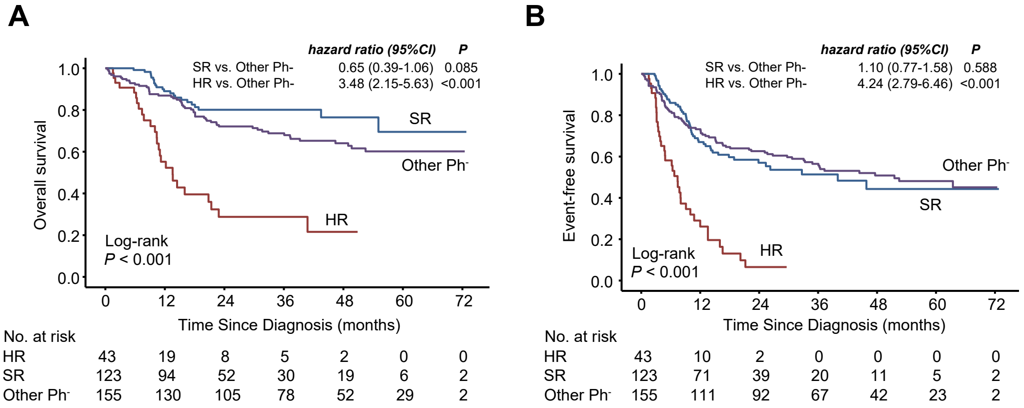

Supplemental Figure 7. OS and EFS of patients stratified by HR and SR subgroups (n=166)

(A) OS and (B) EFS of patients with MRD-positive after induction stratified by HR and SR subgroups; (C) OS and (D) EFS of patients with MRD-negative after induction stratified by HR and SR subgroups.

Abbreviations: MRD, measurable residual disease; HR, high-risk; SR, standard-risk.

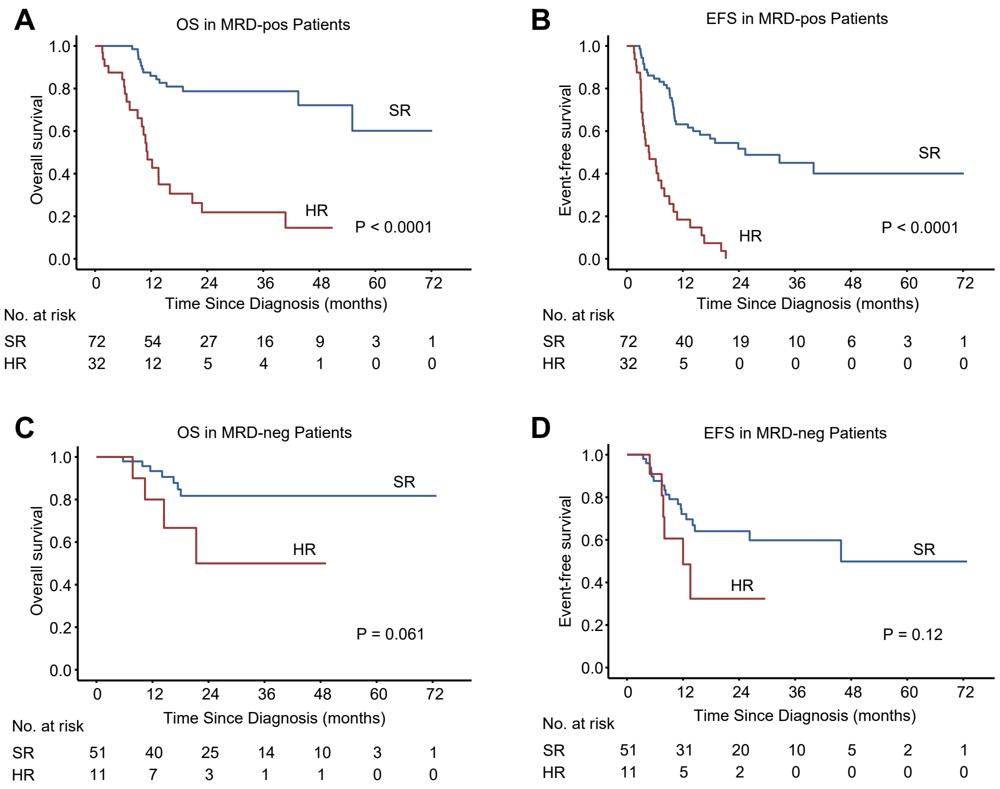

Supplemental Figure 8. Stripplot of WBC count, hemoglobin, platelet count and LDH with embedded value

Missing data were embedded after iteration for 5 times. Blue dots represent the original value. Red dots represent the embedded value.

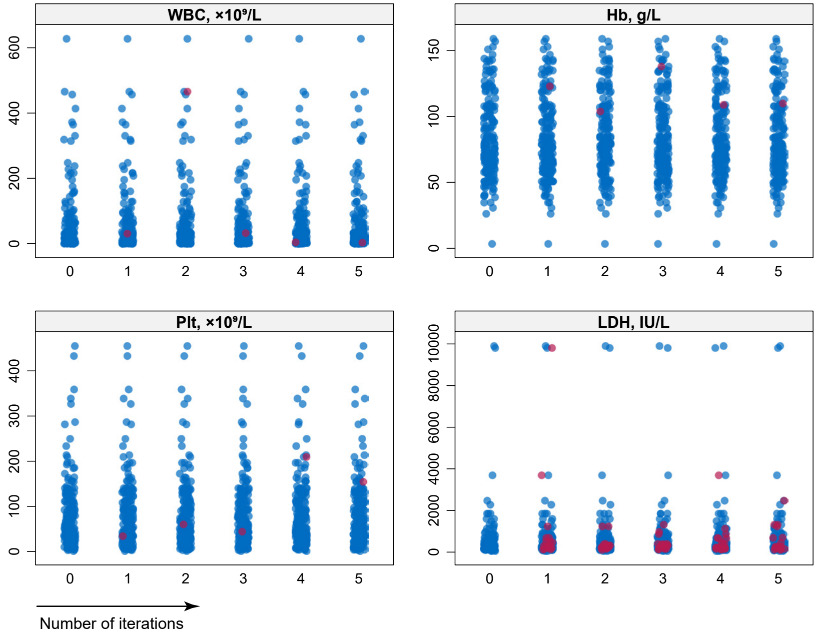

Supplemental Table 1. Baseline characteristics of Ph-like ALL (n = 191) and other Ph-negative patients (n = 155)

Variable	ABL-class	CRLF2	EPOR/JAK2	Other JAK-STAT	Ras	pCRKL/pSTAT5	Ph-like cohort	Other Ph- cohort
**N**	45	52	41	22	17	14	191	155
**Median age, y (range)**	24 (14-64)	28.5 (14-71)	27 (14-55)	22 (14-68)	28 (14-54)	25 (14-49)	26 (14-71)	28 (14-69)
**Sex**								
Female	15 (33.3)	19 (36.5)	18 (43.9)	13 (59.1)	4 (23.5)	5 (35.7)	74 (38.7)	49 (46.7)
Male	30 (66.7)	33 (63.5)	23 (56.1)	9 (40.9)	13 (76.5)	9 (64.3)	117 (61.3)	56 (53.3)
**WBC count, ×10⁹/L**								
＜30	24 (53.3)	35 (67.3)	16 (39.0)	13 (59.1)	12 (75.0)	14 (100.0)	114 (60.0)	108 (70.1)
⩾30	21 (46.7)	17 (32.7)	25 (61.0)	9 (40.9)	4 (25.0)	0 (0.0)	76 (40.0)	46 (29.9)
**Hb, g/L**								
⩾100	11 (24.4)	17 (32.7)	14 (34.1)	7 (31.8)	4 (25.0)	3 (21.4)	56 (29.5)	35 (22.7)
＜100	34 (75.6)	35 (67.3)	27 (65.9)	15 (68.2)	12 (75.0)	11 (78.6)	134 (70.5)	119 (77.3)
**Plt count, ×10⁹/L**								
⩾100	12 (26.7)	14 (26.9)	12 (29.3)	6 (27.3)	3 (18.8)	9 (64.3)	56 (29.5)	59 (38.2)
＜100	33 (73.3)	38 (73.1)	29 (70.7)	16 (72.7)	13 (81.2)	5 (35.7)	134 (70.5)	95 (61.8)
**LDH, IU/L**								
＜600	35 (83.3)	40 (78.4)	28 (80.0)	13 (72.2)	9 (60.0)	7 (58.3)	132 (76.3)	87 (60.8)
⩾600	7 (16.7)	11 (21.6)	7 (20.0)	5 (27.8)	6 (40.0)	5 (41.7)	41 (23.7)	56 (39.2)
***IKZF1* alteration**								
No	24 (58.5)	30 (66.7)	17 (44.7)	10 (58.8)	9 (56.2)	6 (66.7)	96 (57.8)	82 (86.3)
Yes	17 (41.5)	15 (33.3)	21 (55.3)	7 (41.2)	7 (43.8)	3 (33.3)	70 (42.2)	13 (13.7)
**Blinatumomab**								
No	43 (95.6)	49 (94.2)	38 (92.7)	20 (90.9)	16 (94.1)	14 (100.0)	180 (94.2)	151 (97.4)
Yes	2 (4.4)	3 (5.8)	3 (7.3)	2 (9.1)	1 (5.9)	0 (0)	11 (5.8)	4 (2.5)
**CAR-T**								
No	30 (66.7)	30 (57.7)	26 (63.4)	16 (72.7)	12 (70.6)	9 (64.3)	123 (64.4)	143 (92.3)
Yes	15 (33.3)	22 (42.3)	15 (36.6)	6 (27.3)	5 (29.4)	5 (35.7)	68 (35.6)	12 (7.7)
**Allo-HSCT**								
No	14 (31.1)	15 (28.8)	20 (48.8)	11 (50.0)	9 (52.9)	7 (50.0)	76 (39.8)	58 (37.4)
Yes	31 (68.9)	37 (71.2)	21 (51.2)	11 (50.0)	8 (47.1)	7 (50.0)	115 (60.2)	97 (62.6)

Data are median (range) and n (%). 2 patients did not have a documented WBC count, Hb and Plt count. 30 patients did not have a documented LDH. The IKZF1 status of 85 patients was not determined. For targeted drugs in the frontline setting, 32 patients received TKIs (imatinib, dasatinib and orelabrutinib) plus chemotherapy, and 20 patients received ruxolitinib plus chemotherapy. Abbreviations: Ph: Philadelphia chromosome; WBC: white blood cell; Hb: hemoglobin; Plt: platelet; LDH: lactate dehydrogenase; CAR-T, chimeric antigen receptor T; Allo-HSCT: allogeneic stem cell transplantation; SR: standard risk; HR, high risk.

Supplemental Table 2. Univariate analysis and multivariate analysis for EFS (n=166)

Variable		Univariate analysis for EFS		Multivariate analysis for EFS	
		Unadjusted hazard ratio (95% CI)	*P*	Adjusted hazard ratio (95% CI)	*P*
**Age, y**	≥40 vs <40	1.29 (0.75-2.22)	0.364	1.24 (0.70-2.23)	0.461
**WBC count, ×10⁹/L**	≥30 vs <30	1.22 (0.80-1.87)	0.347	1.28 (0.82-1.99)	0.272
IKZF1* plus *CD20	Yes vs No	2.19 (1.36-3.53)	**0.001**	1.33 (0.76-2.32)	0.321
**MRD after inductior**	Neg vs Pos	0.51 (0.32-0.82)	**0.005**	0.59 (0.36-0.98)	**0.041**
**Targeted drugs**	Yes vs No	0.87 (0.54-1.40)	0.557	0.91 (0.55-1.48)	0.693
**CAR-T**	Yes vs No	2.23 (1.57-3.18)	**<0.001**	1.57 (1.01-2.43)	**0.043**
**Allo-HSCT**	Yes vs No	0.60 (0.43-0.85)	**0.004**	0.53 (0.34-0.83)	**0.005**
**Risk stratification**	HR vs SR	4.04 (2.60-6.27)	**<0.001**	3.27 (1.99-5.39)	**<0.001**

MRD negative was defined as less than 1x10-4, and MRD positive was defined as 1x10-4 or higher. Targeted drugs including TKIs (imatinib, dasatinib and orelabrutinib), *JAK2* inhibitor (ruxolitinib). A total of 45 (27.1%) patients received targeted drugs. Abbreviations: CI: Confidence Interval; OS:overall survival; WBC: white blood cell; MRD: measurable residual disease; Neg: negative; Pos: positive; CAR-T, chimeric antigen receptor T; Allo-HSCT: allogeneic stem cell transplantation; HR: high-risk; SR: standard-risk.

Supplemental Table 3. Fusion partner genes of Ph-like ALL identified by RT-PCR or RNA-seq

Ph-like Subtype	Fusion Partner	N
ABL1	*FOXP1*	1
	LSM14A	1
	NUP214	6
	RCSD1	1
	SFPQ	1
	SNX2	1
ABL2	*RCSD1*	4
	ZC3HAV1	1
CSF1R	*MEF2D*	2
PDGFRB	*EBF1*	13
	GTF2I	1
	SMIM3	1
	TEL	2
	TERF2	1
CRLF2	*IGH*	7
	P2RY8	18
EPOR	*IGH*	5
JAK2	*ATF7IP*	1
	BCR	2
	EBF1	1
	IGH	1
	MPRIP	1
	PAX5	8
	PPFIBP1	1
	RABEP1	1
	SMU1	1
	STRBP	1
	TEL	1
	TERF2	4
	ZBTB44	1
	ZEB2	1

Supplemental Table 4. Treatments for patients with or without *IKZF1* alterations (n = 166)

Variable		Non-*IKZF1* alterations	*IKZF1* alterations	Total	*P*
**N**		97	69	166	
**Targeted drugs**	**No**	51 (73.9)	70 (72.2)	121 (72.9)	0.9422
	**Yes**	18 (26.1)	27 (27.8)	45 (27.1)	
**Blinatumomab**	**No**	62 (89.9)	94 (96.9)	156 (94.0)	0.1209
	**Yes**	7 (10.1)	3 (3.1)	10 (6.0)	
**CAR-T**	**No**	40 (58.0)	68 (70.1)	108 (65.1)	0.1469
	**Yes**	29 (42.0)	29 (29.9)	58 (34.9)	
**Allo-HSCT**	**No**	30 (43.5)	34 (35.1)	64 (38.6)	0.3485
	**Yes**	39 (56.5)	63 (64.9)	102 (61.4)	

Data are n (%). Targeted drugs including TKIs (imatinib, dasatinib and orelabrutinib), *JAK2* inhibitor (ruxolitinib). Abbreviations: CAR-T, chimeric antigen receptor T; Allo-HSCT: allogeneic stem cell transplantation; HR: high-risk; SR: standard-risk.

Supplemental Table 5. RR of event occurrence in 166 patients

Variable	RR	95%CI	*P*
**ABL plus *IKZF1***	0.561	0.190-1.654	0.290
CRLF2* plus *IKZF1	**3.545**	1.039-12.104	**0.028**
EPOR/JAK2* plus *IKZF1	**5.614**	1.727-18.248	**0.001**
**JAK-STAT plus *IKZF1***	0.665	0.154-2.879	0.870
**Ras plus *IKZF1***	1.182	0.273-5.117	1.000
pCRKL/pSTAT5* plus *IKZF1	**3.989**	0.889-17.904	**0.048**

RR: Risk Ratio; CI: Confidence Interval

Supplemental Table 6. Treatments for patients within the HR and SR subgroups (n = 166)

Variable		SR	HR	Total	*P*
**N**		97	69	166	
**Targeted drugs**	**No**	86 (69.9)	35 (81.4)	121 (72.9)	0.2084
	**Yes**	37 (30.1)	8 (18.6)	45 (27.1)	
**Blinatumomab**	**No**	117 (95.1)	39 (90.7)	156 (94.0)	0.4982
	**Yes**	6 (4.9)	4 (9.3)	10 (6.0)	
**CAR-T**	**No**	81 (65.9)	27 (62.8)	108 (65.1)	0.8596
	**Yes**	42 (34.1)	16 (37.2)	58 (34.9)	
**Allo-HSCT**	**No**	42 (34.1)	22 (51.2)	64 (38.6)	0.0732
	**Yes**	81 (65.9)	21 (48.8)	102 (61.4)	

Data are n (%). Targeted drugs including TKIs (imatinib, dasatinib and orelabrutinib), *JAK2* inhibitor (ruxolitinib). Abbreviations: CAR-T, chimeric antigen receptor T; Allo-HSCT: allogeneic stem cell transplantation; HR: high-risk; SR: standard-risk.
